# Photochemical enhancement of PD-L1-SAP immunotoxin efficacy in non-small cell lung cancer cell lines

**DOI:** 10.3389/fimmu.2026.1750003

**Published:** 2026-03-13

**Authors:** Magdaléna Kozlíková, Inger Kristine Fjeldskaar Aukrust, Monika Rohlíčková, Miloslav Macháček, Kristian Berg, Anette Weyergang, Pål Kristian Selbo

**Affiliations:** 1 Department of Biochemical Sciences, Faculty of Pharmacy in Hradec Králové, Charles University, Hradec Králové, Czechia; 2 Department of Radiation Biology, Institute for Cancer Research, The Norwegian Radium Hospital, Oslo University Hospital, Oslo, Norway

**Keywords:** atezolizumab, endosomal escape, immune checkpoint inhibitors, intracellular drug delivery, non-small cell lung cancer, PD-L1-targeted immunotoxin, photochemical internalization, photodynamic therapy

## Abstract

**Introduction:**

Resistance to immune checkpoint inhibitors (ICIs) targeting the programmed cell death protein 1/programmed death ligand 1 (PD–1/PD–L1) axis remains a major obstacle in non–small cell lung cancer (NSCLC).

**Materials and methods:**

To address this, we investigated photochemical internalization (PCI), a light–controlled endosomal escape technology, as a strategy to enhance intracellular delivery and efficacy of a PD–L1–targeted immunotoxin (anti–PD–L1–SAP).

**Results:**

NSCLC cell lines with high (NCI–H1975) and low (A549) PD–L1 expression were subjected to PCI, resulting in a pronounced increase in cytotoxicity with picomolar potency (30 pM), while A549 cells required a higher dose (1000 pM) for a similar effect. Specificity was confirmed via receptor blockade and non–targeted controls. Confocal microscopy demonstrated lysosomal and endosomal localization of anti–PD–L1–SAP, and flow cytometry showed time–dependent intracellular accumulation, consistent with PCI’s requirement for endosomal sequestration prior to light–induced release. Importantly, co–treatment with the immune checkpoint inhibitor atezolizumab (Tecentriq^®^) reduced PCI efficacy in PD–L1^^high^^ cells, underscoring the importance of receptor accessibility.

**Conclusions:**

These findings demonstrate that PCI enhances delivery and activity of PD–L1–targeted biologics and may help overcome resistance mechanisms. Overall, PCI expands the therapeutic window of PD–L1–targeted immunotoxins and may complement current immunotherapies, supporting further preclinical evaluation in NSCLC.

## 1 Introduction

Targeting the PD-1/PD-L1 axis with immune checkpoint inhibitors (ICIs), has transformed the outcome of cancer therapy for many indications, including treatment of non-small cell lung cancer (NSCLC). These agents restore anti-tumor immunity by reactivating exhausted T-cells. PD-1, an inhibitory receptor on activated T-cells, binds to PD-L1, which is frequently overexpressed on tumor cells and immunosuppressive cells within the tumor microenvironment (TME). This interaction suppresses cytotoxic T-cell activity and facilitates immune escape, making PD-1/PD-L1 a key target for immunotherapy. The clinical efficacy of immune checkpoint inhibitors (ICIs) is well established, leading to the approval of anti-PD-1 agents (e.g., nivolumab, pembrolizumab) and anti-PD-L1 agents (e.g., atezolizumab, durvalumab) for the treatment of multiple malignancies, including NSCLC (1, 2). However, a substantial proportion of patients fail to respond or develop resistance over time. Primary resistance may arise from tumor-intrinsic factors such as defective antigen presentation (e.g., B2M loss), mutations in JAK1/2 affecting interferon-γ signaling, or activation of the WNT/β-catenin pathway that impairs T-cell infiltration (3, 4). Acquired resistance can result from adaptive immune escape mechanisms, including PD-L1 upregulation and T-cell exhaustion (5). The immunosuppressive tumor microenvironment (TME), rich in regulatory T-cells (Tregs), myeloid-derived suppressor cells (MDSCs), tumor-associated macrophages (TAMs), and alternative checkpoints like TIM-3 and LAG-3, further limits ICI efficacy (6). Additionally, low tumor mutational burden (TMB) and poor neoantigen presentation contribute to reduced responsiveness (7). The multifactorial nature of resistance to ICIs underscores an urgent need for development of novel strategies to overcome this challenge. Emerging combination approaches including dual checkpoint blockades, targeted therapies and innovative drug delivery systems aim to enhance immune activation and improve treatment outcomes. Continued exploration of these strategies is essential to overcome current limitations and expand the clinical benefit of ICIs across diverse patient populations.

One such emerging strategy involves photochemical internalization (PCI), a light-controlled drug delivery technology designed to enhance the intracellular delivery of macromolecules that are otherwise trapped in endosomes and lysosomes. PCI builds on the principles of photodynamic therapy (PDT), using an amphiphilic photosensitizer such as fimaporfin (TPCS_2a_), that preferentially accumulates in endolysosomal compartments. Upon light activation, the PCI photosensitizer induces the generation of reactive oxygen species (ROS), which compromise the integrity of endolysosomal membranes. This membrane disruption facilitates the release of the therapeutic payload into the cytosol, thereby enhancing intracellular delivery and therapeutic efficacy (8, 9). In contrast to PDT, which induces cell death through ROS-mediated damage directly, PCI aims to facilitate ROS-induced endosomal destabilization and subsequent improved intracellular drug delivery, thereby enhancing the efficacy and specificity of biologics.

Ribosome inactivating proteins (RIPs), particularly type I RIPs such as saporin and gelonin, represent attractive payloads for delivery by PCI. These proteins enzymatically inactivate ribosomes, leading to irreversible inhibition of protein synthesis and cell death. Saporin has been shown to enter the cell by endocytosis. However, due to its inability to escape endosomes, the RIP efficacy is limited unless combined with an endosome escape strategy such as PCI (10, 11). Upon conjugation with tumor-specific monoclonal antibodies, RIPs can be targeted to cancer-associated antigens, improving both selectivity and cytotoxic potential (12).It has been demonstrated that PD-L1 undergoes internalization via endocytosis (13). Moreover, we recently demonstrated that the PD-L1-targeting mAb atezolizumab (Tecentriq) is taken up into NCLC cells and is transported via CD63-positive organelles to the lysosomes (14). Anti-PD-L1-SAP, a novel immunotoxin designed to target PD-L1-expressing cells, has previously shown promise as a PCI-compatible agent in breast cancer models (15), further supporting its potential utility in NSCLC.

Preclinical studies have shown that PCI significantly enhances the efficacy of macromolecular drugs, including both chemotherapeutics and toxin-conjugated antibodies (16–19). Fimaporfin-based PCI has also been tested in several clinical trials. In the first phase I trial (ClinicalTrials.gov identifier: NCT00993512), PCI with bleomycin demonstrated a favorable safety and tolerability profile. Moreover, it induced significant antitumor responses in patients with squamous cell carcinoma of the head and neck, as well as in some cases of advanced or recurrent cutaneous or subcutaneous malignancies involving the torso or upper limbs such as sarcoma, eccrine (adnexal) carcinoma, and chemoresistant ductal breast carcinoma (20). In the second phase I trial (ClinicalTrials.gov identifier: NCT01900158, PCI using fimaporfin and gemcitabine was also found to be safe and showed promising efficacy in patients with inoperable perihilar cholangiocarcinoma (CCA). The phase I study reported disease control rates, two complete responses, and extended survival, supporting PCI as a potential enhancement to standard chemotherapy in this patient group (21). However, the follow-up Phase II trial (RELEASE, ClinicalTrials.gov identifier: NCT04099888) was terminated due to promising data from the TOPAZ-1 Phase III trial (ClinicalTrials.gov identifier: NCT03875235), which demonstrated adding immunotherapy with durvalumab, a human anti-PD-L1 monoclonal antibody (mAb), to standard chemotherapy increased overall survival in advanced biliary tract cancer. This changed the standard of care for inoperable CCA (22). Finally, a phase I trial evaluated PCI for HPV peptide vaccination, establishing its feasibility as an immunomodulatory platform (ClinicalTrials.gov identifier: NCT02947854) (23).

In this study, we provide *in vitro* proof–of–concept that photochemical internalization can potentiate the intracellular delivery and cytotoxic activity of a PD–L1–targeted immunotoxin in NSCLC. To determine how effectively PCI enhances selective targeting of PD–L1–expressing cells, we investigated two NSCLC models that differ markedly in PD–L1 abundance, NCI–H1975 (PD-L1^high^) and A549 (PD-L1^low^). We assessed immunotoxin cytotoxicity, PDT and PCI efficacy, and performed detailed analyses of PD–L1 binding, internalization dynamics, and subcellular localization of the PD–L1–targeting monoclonal antibody atezolizumab.

## 2 Material and method

### 2.1 Cell culture

The human NSCLC cell lines NCI-H1975 (CRL-5908) and A549 (CCL-185) were obtained from the American Type Culture Collection (ATCC, USA). NCI-H1975 cells were cultured in RPMI-1640 medium (GIBCO, Thermo Fisher Scientific, USA, cat. no. 11875093), while A549 cells were incubated in Ham’s F-12K (Kaighn’s) medium (GIBCO, Thermo Fisher Scientific, USA, cat. no. 21127022). Media were supplemented with 10% (v/v) fetal bovine serum (GIBCO, Thermo Fisher Scientific, USA, cat. no. A5670701), 100 U/mL penicillin and 100 µg/mL streptomycin (Sigma-Aldrich, USA, cat. no. P0781). Cells were maintained in an incubator at 37 °C with a humidified atmosphere of 5% CO_2_ in air.

### 2.2 Drugs and cytotoxicity

The photosensitizer disulfonated tetraphenyl chlorin (TPCS_2a_, also known as fimaporfin) was obtained from PCI Biotech AS (Norway). The TPCS_2a_ stock solution was prepared at a concentration of 0.35 mg/mL in polysorbate 80 with 2.8% mannitol, 50 mM Tris (pH 8.5) and stored in dark at 4 °C. Atezolizumab (Tecentriq, Roche) was provided by the Hospital Pharmacy at Radiumhospitalet, Oslo University Hospital.

### 2.3 PCI of anti-PD-L1-SAP

The immunotoxin PD-L1-SAP (Anti-PD-L1-SAP, cat. no: IT-45 and KIT-45) was obtained from Advanced Targeting Systems (ATS, USA). PD-L1-SAP is a combination of a mouse monoclonal antibody against human PD-L1 and secondary conjugate streptavidin-saporin with the size of 296 kDa. Biotinylated mouse IgG and the secondary conjugate streptavidin-saporin A (both from ATS) were used as an untargeted immunotoxin control (BIgG–SAP, cat. no. IT-74).

NCI-H1975 (4 × 10 (3) cells/well) and A549 (6 × 10^3^ cells/well) cells were seeded into 96-well plates (Nunclon, Thermo Fisher Scientific, Denmark, cat. no. 734-2097) and were left to grow overnight. Cells were then incubated with TPCS_2a_ and anti-PD-L1-SAP for 18 h. The concentration of TPCS_2a_ was 0,2 µg/mL and 0,5 µg/mL in NCI-H1975 and A549, respectively while the concentration of anti PD-L1-SAP was 30, 100 and 1–000 pM for NCI-H1975 cell line and 1000 pM for A549 cell line. The PD-L1-SAP concentrations were determined from initial evaluations of immunotoxin potency in both cell lines. Different dose ranges were required for PD-L1^high^ (NCI-H1975) and PD-L1^low^ (A549) cells, as the cytotoxicity of anti-PD-L1-SAP correlated with PD-L1 expression levels, requiring adjustment of concentrations to achieve measurable and comparable biological effects. Two experimental workflows were applied. In Workflow I, cells were incubated with both TPCS_2a_ and anti–PD–L1–SAP for 18 h, washed twice with fresh medium, and subsequently incubated for 4 h in drug–free medium to allow redistribution/removal of membrane–associated TPCS_2a_ before irradiation. In Workflow II, cells were first incubated with TPCS_2a_ alone for 18 h; during the subsequent 4 h chase period, anti–PD–L1–SAP was added to enable its internalization prior to light exposure. This sequential protocol allowed us to assess how the timing of immunotoxin uptake relative to photosensitizer activation influences PCI efficacy. The LumiSource lamp (PCI Biotech, Norway) was used as a light source to activate the photosensitizer in all experiments (PDT and PCI). This is a broadband blue lamp (emission range of λ: 400–500 nm) with an emission peak at 435 nm (4 x 18 W Osram L 18/67, Blue). The irradiance of the sample surface was measured at 10 mW/cm^2^. TPCS_2a_ exhibits a major absorption peak at λ = 420 nm. Based on these parameters, a light exposure of 0.5, 1, 1.5, 2, 2.5 and 3 min delivers a light dose of 0.3, 0.6, 0.9, 1.2, 1.5 and 1.8 J/cm², respectively.

### 2.4 Blocking PD-L1 receptors with atezolizumab before PCI of anti-PD-L1-SAP

To investigate whether the clinically approved anti-PD-L1 antibody atezolizumab (Tecentriq^®^) modulates PCI efficacy, we applied it in combination with Workflow II. The aim was to determine whether receptor blockade by atezolizumab enhances or inhibits the PCI-mediated cytotoxicity of anti-PD-L1-SAP in A549 and NCI-H1975 lung cancer cell lines. Cells were seeded as described above and preincubated with TPCS_2a_ for 18 h to allow photosensitizer accumulation. Subsequently, the cells were washed with fresh medium and preincubated for 30 min with atezolizumab (100 nM) to block PD-L1 receptors. The medium was then replaced with fresh medium containing both atezolizumab (100 nM) and anti-PD-L1-SAP (1–000 pM), and cells were incubated for an additional 4 h (chase period) to allow uptake. Before light exposure, the cells were washed again and replenished with fresh medium.

### 2.5 Measurement of cytotoxic responses by the MTS assay

Metabolic activity, used as a proxy for cell viability was determined 48 h after irradiation using the MTS assay (Promega, cat. no G1111), according to the manufacturer’s instructions. Briefly, 0.33 mg/mL of MTS assay was added to 100 µl of fresh medium in each well and incubated for 2 h at 37 °C. Plates were then briefly shaken, and absorbance was measured at 490 nm using a microplate reader (Biotek Instruments Inc., USA).

### 2.6 Confocal microscopy and quantitative analysis

PCI is dependent on endo/lysosomal accumulation of the therapeutic of interest. Thus, PCI of PD-L1-SAP is dependent on trafficking to these organelles. Binding, uptake and subcellular localization of PD-L1 were investigated in both NCI-H1975 and A549 cells by confocal microscopy. Cells were seeded at a density of 1 × 10^5^ cells per Petri dish (35 mm) suitable for confocal microscopy (Willco Well, Netherlands, cat. no. HBST-3522). Cells were incubated with biotinylated anti-human CD274 (B7-H1, PD-L1) antibody (BioLegend, USA, cat. no. 329704), and Alexa Fluor 647-conjugated Streptavidin (BioLegend, USA, cat. no. 405237) at dilutions of 1:400 and 1:265, respectively, for 0.5, 1, 2, 4, 8 and 18 h. Cells were then washed twice with fresh medium and incubated with 0.4 μM LysoTracker Blue DND-22 (Molecular Probes, Thermo Fisher Scientific, USA, cat. no. L7525) and 0,1× CellMask Green Plasma Membrane Stain (Molecular Probes, Thermo Fisher Scientific, USA, cat. no. C37608) for 20 min at 37 °C. After staining, cells were washed twice, fresh medium was added, and samples were placed into a stage-top incubator (Okolab) of Nikon A1+ laser scanning confocal system (Nikon, Japan) operated via NIS Elements AR 5.4 software (Laboratory Imaging, Czech Republic). Image acquisition was performed using 405, 488 and 640 nm lasers with 60× oil immersion objective lens. Confocal zoom was set to1.51× under Nyquist sampling conditions), with a resolution of 1024×1024 pixels (px) (0.14 µm/px) and 8× line averaging. The pinhole diameter was set to 28.1 µm, and one confocal plane per field of view was captured. Colocalization of anti-PD-L1-Alexa647 with either cytoplasmic membrane or lysosomes was quantified using Pearson’s correlation coefficient (PCC) and Mander’s Overlap coefficient (MOC).

PCC measures the linear correlation between pixel intensities of two fluorophores ranging from -1 to 1, where 1 indicates perfect positive correlation, 0 no correlation, and -1 perfect negative correlation. It is sensitive to intensity variations and not affected by background or signal magnitude. MOC assesses the spatial overlap between two signals, independent of intensity correlation, with values ranging from 0 (no overlap) to 1, with 1 (complete overlap).

### 2.7 Flow cytometry

NCI-H1975 and A549 cells were seeded at a density of 1×10^5^ cells/well in 12-well tissue culture plates (TPP Techno Plastic Products, Switzerland, cat. no. 92012). The cells were incubated simultaneously with biotinylated antibody against human CD274 (B7-H1/PD-L1) (BioLegend, USA, cat. no. 329704; dilution 1:400) and streptavidin conjugated with Alexa Fluor 647 (BioLegend, USA, cat. no. 405237; dilution 1:256). Incubation times included 0.5, 1, 2, 4, 8 and 18 h. An additional experimental condition consisted of an 18 h incubation followed by a 4 h chase period. After incubation, the medium was removed, and cells were washed with cold PBS. Cells were detached using Accutase (Thermo Fisher Scientific, USA, cat. no. 00-4555-56) for 3 min at room temperature, followed by resuspension in cold PBS containing 0.5% BSA (Capricorn, Germany, cat. no. BSA-1U). Samples were immediately analyzed using a Sony SA6800 spectral flow cytometer (Sony Biotechnology). Samples not immediately measured were kept on ice. To distinguish dead cells, cells were stained with CellTox™ Green Cytotoxicity Assay (Promega, USA, cat. no. G8741) at a dilution of 1: 6000. The following controls were included: Unstained cells; cells stained with CellTox Green; cells stained with biotinylated anti-human CD274 and Alexa Fluor 647Streptavidin; and cells stained with both CellTox Green and biotinylated anti-human CD274 and Alexa Fluor 647–conjugated Streptavidin.

### 2.8 Statistics

Statistical analysis was performed using GraphPad Prism version 10.5.0 for Windows (GraphPad Software, Boston, MA, USA), PDT vs. PCI. An unpaired t-test with Welch’s correction was used to assess whether the difference between the PDT vs. PCI (PDT + anti-PD-L1-SAP) response was statistically significant or not. Results are expressed as mean ± standard deviation (SD). All experiments were performed in at least three independent biological replicates. Within each independent experiment, conditions were plated in technical triplicates (three wells per treatment).

## 3 Results

### 3.1 Phototoxicity of TPCS_2a_ on lung cancer cell lines

**Figure 1:**
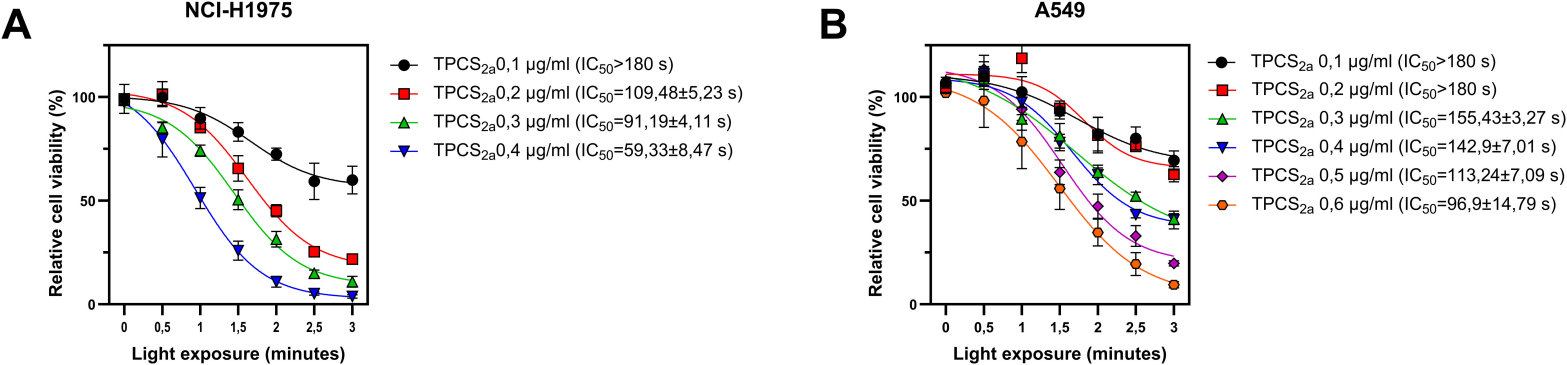
Phototoxicity of TPCS_2a_-PDT in PD-L1^high^ NCI-H1975 cells, **(A)** and PD-L1^low^ A549, **(B)** cell lines. Cell viability was assessed 48 h post light exposure (3 min = 1.8 J/cm²). Data represents mean ± SD of three independent experiments (n = 3).

To establish optimal conditions for PCI, we first evaluated the phototoxicity of TPCS_2a_ in the NSCLC cell lines NCI-H1975 and A549. Initial PDT experiments were conducted using a range of TPCS_2a_ concentrations (0.1–0.6 µg/mL) and irradiation times from 0 to 3 min (**Figures 1A, B**). The PD-L1^high^ NCI-H1975 cells exhibited greater sensitivity to PDT compared to the PD-L1^low^ A549 cells, as reflected by reduced viability at lower concentrations and shorter irradiation times, and reduced IC_50_ values on NCI-H1975 cells compared to A549 cells (**Figures 1A, B**). Based on these results, TPCS_2a_ concentrations of 0.2 µg/mL for NCI-H1975 and 0.5 µg/mL for A549 were selected for subsequent PCI experiments to ensure sufficient photochemical effect while minimizing non-specific cytotoxicity.

### 3.2 Cytotoxicity of anti-PD-L1-SAP and non-specific BIgG-SAP immunotoxin

To determine appropriate dosing for PCI and measure baseline toxicity, we next assessed the cytotoxicity of the PD-L1-targeting immunotoxin (anti-PD-L1-SAP) and a non-specific saporin-conjugated antibody (BIgG-SAP) in the absence of fimaporfin and light. Although similar experimental conditions were previously reported by Wong and Selbo (15) on breast cancer cell lines, the different cancer type and cell lines used in this study necessitated independent validation.

**Figure 2:**
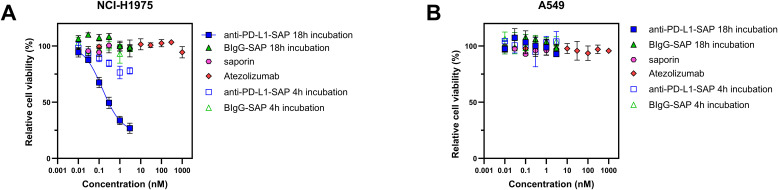
Cytotoxicity of anti-PD-L1-SAP, BIgG-SAP, saporin, and atezolizumab in the absence of photosensitizer and light in two NSCLC cell lines. **(A)** NCI-H1975 cells and **(B)** A549 cells. In all experiments, cell viability was assessed after 48 h incubation. Data represents mean ± SD of three independent experiments (n = 3).

The non-targeted immunotoxin BIgG-SAP showed no reduction in viability in either cell line across a concentration range of 1 pM to 3000 pM (**Figures 2A, B**). Similarly, free saporin exhibited no reduction in viability under the same conditions (**Figure 2B**). In the PD-L1^low^ A549 cell line, anti-PD-L1-SAP also failed to reduce viability at indicated concentrations. In contrast, the PD-L1^high^ NCI-H1975 cell line displayed a dose-dependent reduction in viability, with 31% and 63.5% decreases observed at 100 pM and 1000 pM, respectively. The IC_50_ value of anti-PD-L1-SAP for the H1975 cell line is 0.29 ± 0.09 nM.

Further viability assessments following a shorter 4-h incubation of the immunotoxins confirmed these findings as no significant reduction in viability was observed in either cell line treated with BIgG-SAP or saporin alone (**Figures 2A, B**), while anti-PD-L1-SAP induced modest reduction in viability of 11% and 24% in NCI-H1975 cells at 100 pM and 1000 pM concentrations, respectively.

### 3.3 Cytotoxic effects of PCI of anti-human PD-L1-SAP

**Figure 3:**
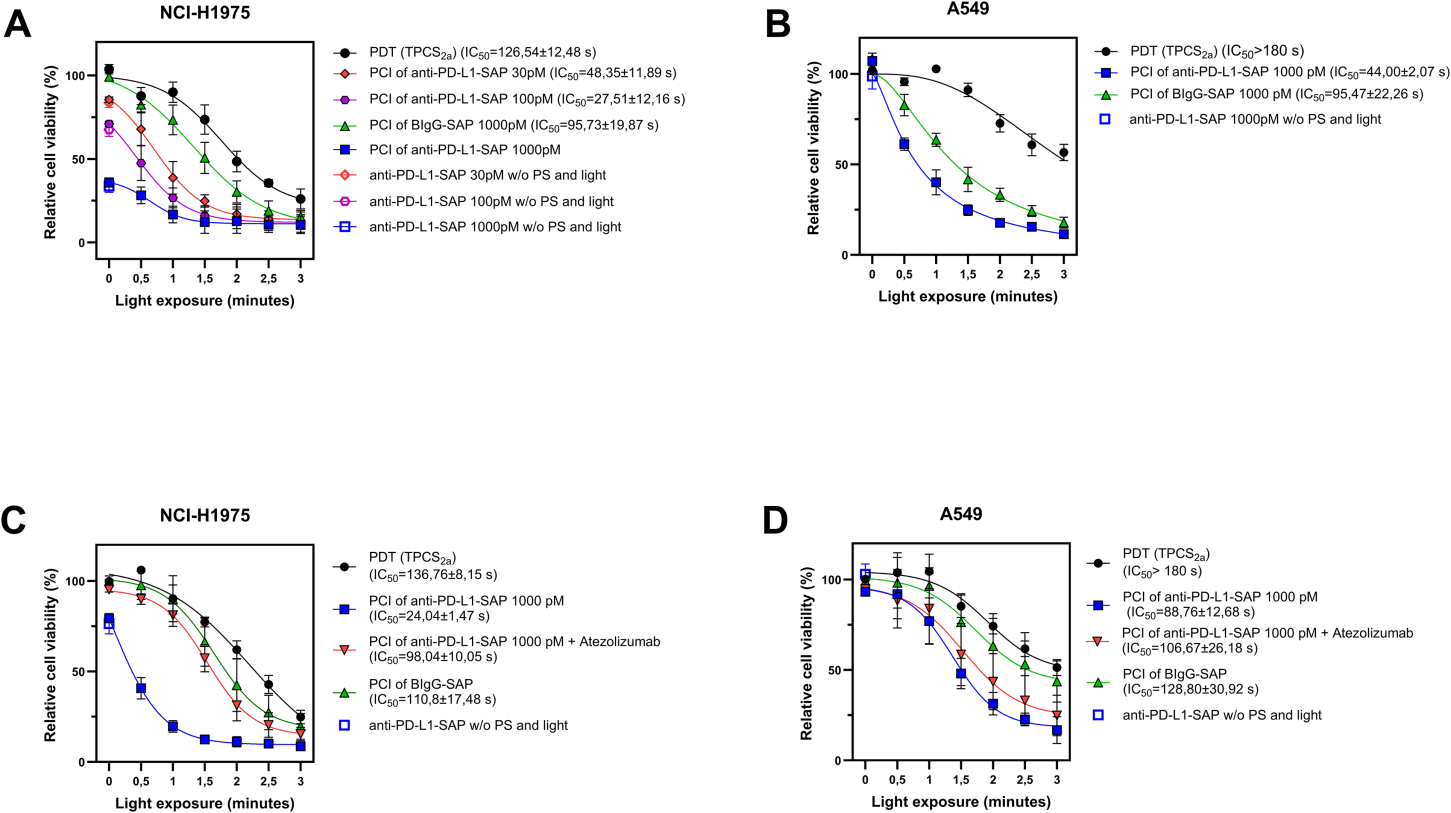
Cytotoxic effects of PCI of anti-human PD-L1-SAP in lung cancer cell lines. PCI and PDT Workflow I on **(A)** NCI-H1975 cells and **(B)** A549. PCI and PDT in NCI-H1975 **(C)** and A549 **(D)** cells using Workflow II. In all experiments, cell viability was assessed 48 h post irradiation (3 min = 1.8 J/cm²). Data represents mean ± SD of three independent experiments (n = 3). Exact p-values for all PCI experiments (PDT vs. PCI with anti-PD-L1-SAP) are shown in **Supplementary Table 1**.

Based on the results from the preceding experiments, we next evaluated the efficacy of PCI for targeted delivery of the PD-L1–specific immunotoxin anti–PD-L1–SAP. PCI efficacy was studied with two different protocols, one with 18 h incubation of the targeting toxins and one with 4 h incubation as described in the M&M section. Using Workflow I (co-incubation of TPCS_2a_ and anti–PD-L1–SAP for 18 h followed by a 4 h chase period), a significant increase in cytotoxicity was observed in PD-L1^high^ NCI-H1975 cells at 30 and 100 pM anti–PD-L1–SAP, compared to the non-targeted control BIgG–SAP (**Figure 3A**). However, in the PD-L1^high^ NCI-H1975 cell line, the immunotoxin alone reduced cell viability, resulting in decreases of approximately 17% and 33% at concentrations of 30 pM and 100 pM, respectively. In contrast, PD-L1^low^ A549 cells required a higher concentration (1000 pM) of anti–PD-L1–SAP to achieve a comparable PCI effect (**Figure 3B**).

Using Workflow II (pre-incubation with TPCS_2a_ for 18 h followed by a 4 h incubation with anti–PD-L1–SAP), PCI efficacy was also achieved in NCI-H1975 cells at 1000 pM anti–PD-L1–SAP (**Figure 3C**), demonstrating that a shorter immunotoxin exposure is sufficient when using a higher concentration. However, in A549 cells, this protocol resulted in a 2× lower PCI effect compared to Workflow I based on IC_50_ values (**Figure 3D**). Statistical comparisons between PDT and PCI treatments are provided in **Supplementary Table 1**.

### 3.4 Modulation of PCI efficacy by atezolizumab

In the PD-L1^high^ NCI-H1975 cell line, co-treatment with atezolizumab significantly reduced the PCI effect during the initial 90 seconds of irradiation (p = 0,005) as shown in **Figure 3C** (red curve). The resulting cytotoxicity profile closely resembled that of PDT alone, suggesting that atezolizumab competitively blocked PD-L1 binding sites, thereby limiting immunotoxin uptake and intracellular delivery. In contrast, no significant reduction in PCI efficacy was observed in A549 cells (**Figure 3D**), indicating that the lower PD-L1 expression may diminish the impact of receptor blockade under these conditions.

Statistical comparisons for all PCI + atezolizumab experiments (PDT vs. PCI with anti–PD-L1–SAP + atezolizumab) are provided in **Supplementary Table 1**.

### 3.5 Intracellular localization and trafficking of anti-PD-L1

Fluorescence imaging showed progressive internalization of PD-L1 from the plasma membrane into intracellular compartments. PD-L1 was initially detected at the cell surface and gradually accumulated in acidic vesicles. This approach provided detailed insight into the temporal and spatial dynamics of PD-L1 internalization and lysosomal trafficking in NSCLC cells with distinct PD-L1 expression profiles.

**Figure 4:**
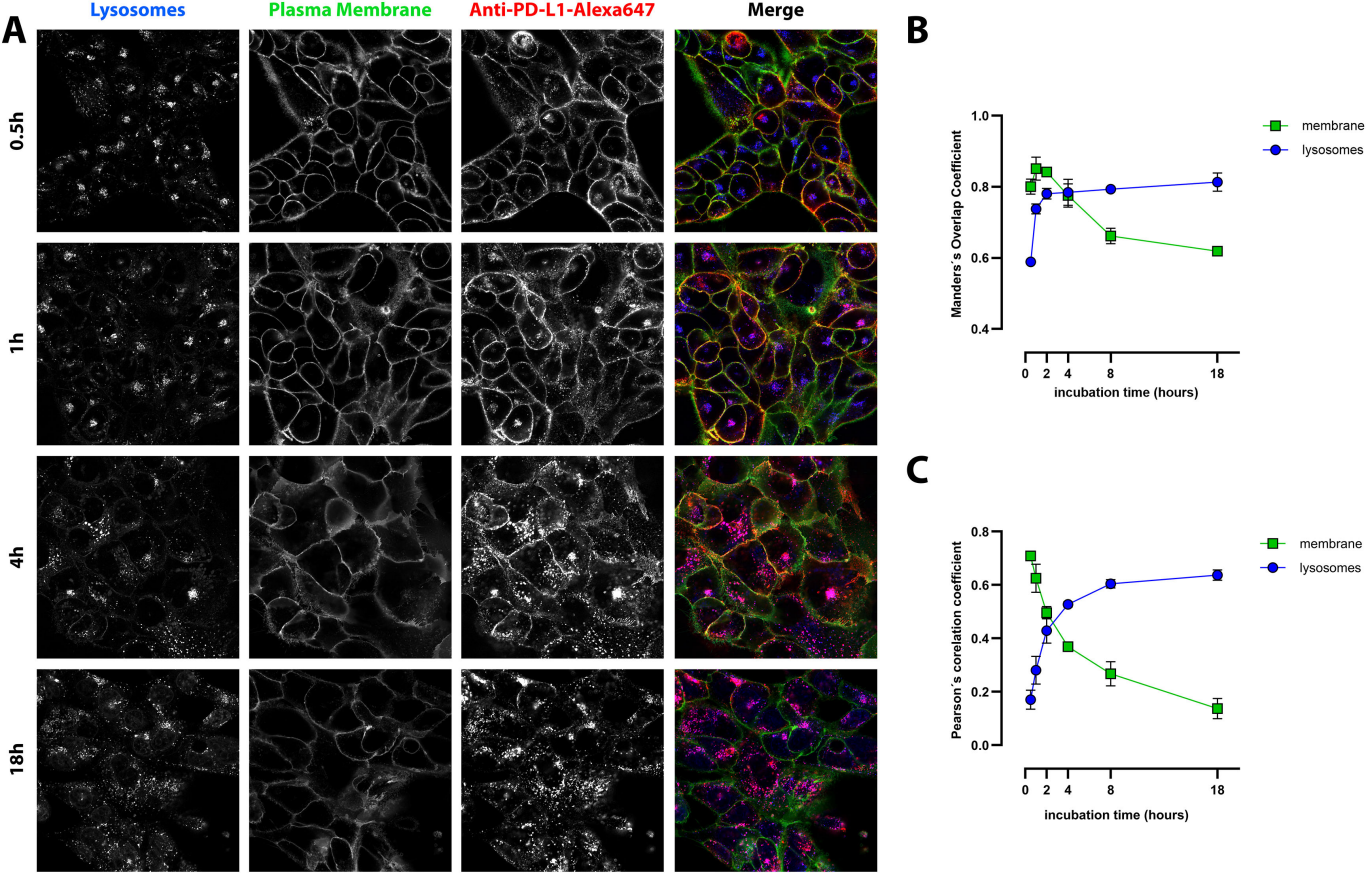
Cell membrane binding and intracellular trafficking of anti-PD-L1-AlexaFluor647 in PD-L1^high^ NCI-H1975 cells. **(A)** Representative photomicrographs of selected time points are shown (for the remaining time points see **Supplementary Figure 1**). Red: anti-PD-L1-AlexaFluor647; green: plasma membrane; blue: LysoTracker blue (late endosomes and lysosomes), pink – merge of LysoTracker blue and anti-PD-L1-AlexaFluor647, yellow/orange – merge of plasma membrane and anti-PD-L1-AlexaFluor647. Scale bar 20 µm. **(B, C)** Quantification of anti-PD-L1-AlexaFluor647 colocalization with lysosomes and plasma membrane using Mander’s overlap coefficient **(B)** and Pearson’s correlation coefficient (**C**). The results are mean ± SD of three independent experiments.

At early time points (0.5–2 h), anti-PD-L1-Alexa647 was predominantly localized at the plasma membrane in PD-L1^high^ NCI-H1975 cells, as indicated by the orange signal resulting from the overlap of membrane (green) and anti-PD-L1-Alexa647 (red)fluorescence. Over time (2–18 h), the PD-L1 signal progressively shifted to intracellular compartments, showing increasing colocalization with lysosomes, visualized as a pink signal from the overlap of blue and red fluorescence (**Figure 4A**). This temporal pattern suggests active internalization of PD-L1 and subsequent trafficking toward lysosomal compartments.

Quantitative analysis using Pearson’s and Mander’s colocalization coefficients supported these observations. At 30 min, PD-L1 showed strong colocalization with the plasma membrane, with a Pearson’s correlation coefficient of 0.71 ± 0.01, which declined to 0.14 ± 0.04 after 18 h (**Figure 4C**). Similarly, Mander’s overlap coefficient for membrane colocalization decreased from 0.8 ± 0.02 to 0.62 ± 0.01 over the same period (**Figure 4B**).

In contrast, colocalization of PD-L1 with lysosomes increased over time. The Pearson’s coefficient rose from 0.17 ± 0.04 at 30 min to 0.64 ± 0.02 at 18 h, while Mander’s overlap coefficient increased from 0.59 ± 0.01 to 0.81 ± 0.03. These results indicate progressive internalization and lysosomal trafficking of PD-L1, highlighting a dynamic intracellular fate that may influence immune signaling and the therapeutic efficacy of PD-L1–targeted agents.

**Figure 5:**
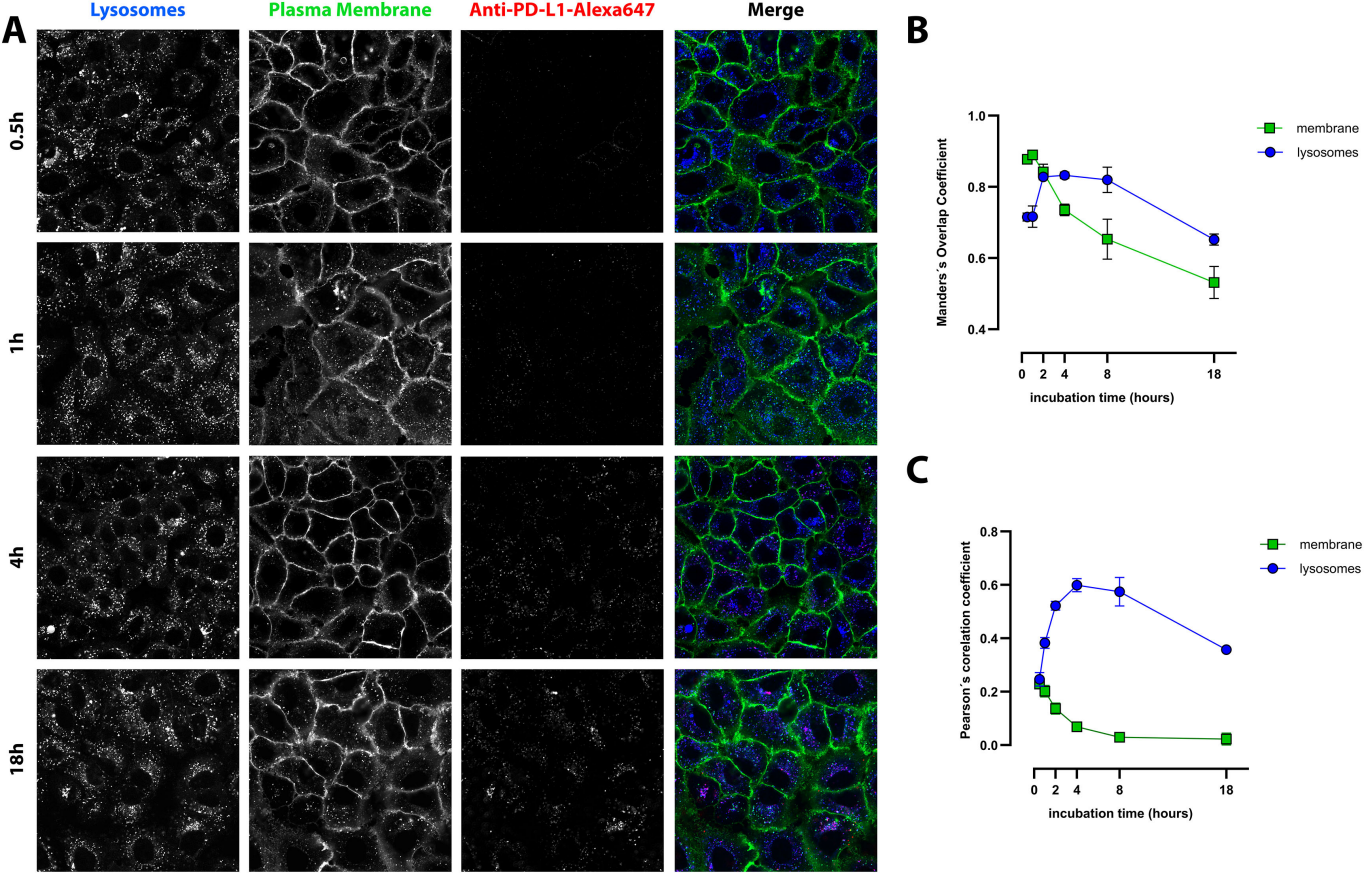
Intracellular localization and trafficking of anti-PD-L1-AlexaFluor647 in PD-L1^low^ A549 cells. **(A)** Representative photomicrographs of selected time points (for the remaining time points see **Supplementary Figure 2**). Red: anti-PD-L1-AlexaFluor647; green: plasma membrane; blue: LysoTracker blue (late endosomes and lysosomes), pink – merge of LysoTracker blue and anti-PD-L1-AlexaFluor647. Scale bar 20 µm. **(B, C)** Quantitative analysis of PD-L1 colocalization with lysosomes and plasma membrane using Pearson’s correlation coefficient **(B)** and Mander’s overlap coefficient **(C)**. The data represent mean ± SD of three independent experiments (n = 3).

In the PD-L1^low^ A549 cell line, PD-L1 trafficking did not follow a strictly progressive pattern of lysosomal accumulation over time (**Figure 5A**). Colocalization analysis using Pearson’s correlation coefficient revealed a moderate initial association with lysosomes (0.25 ± 0.03 at 30 min), which peaked at 4 h (0.60 ± 0.02) before declining to 0.36 ± 0.03 at 18 h (**Figure 5C**). In contrast, colocalization with the plasma membrane steadily decreased over time, with Pearson’s values dropping from 0.23 ± 0.02 at 30 min to 0.02 ± 0.02 at 18 h, indicating progressive internalization of PD-L1 from the cell surface.

Similar trends were observed using Mander’s overlap coefficient. Lysosomal colocalization peaked at 0.83 ± 0.01 at 4 h and declined to 0.65 ± 0.02 at 18 h (**Figure 5B**), while membrane colocalization steadily decreased throughout the time course. These data suggest a more rapid lysosomal degradation of PD-L1 in low-expressing cells, distinct from the trafficking pattern observed in PD-L1^high^ cells.

Confocal microscopy confirmed that NCI-H1975 cells express significantly higher levels of PD-L1 than A549 cells, as evidenced by stronger Alexa647 fluorescence intensity across all time points. In NCI-H1975 cells, strong colocalization with the plasma membrane was observed during the first 2 h, whereas in A549 cells, membrane colocalization was weak and declined to nearly undetectable levels.

Lysosomal colocalization in NCI-H1975 cells at 4 and 18 h also supports the PCI results, indicating that PD-L1-sap was localized to lysosomes at these time points and therefore subjected to PCI release and activation. In A549 cells, lysosomal colocalization peaked at 4 h, corresponding to the time of maximal internalization. However, PCI results suggest that due to lower PD-L1 levels, this was insufficient to trigger an effective response during the initial minutes of illumination. Notably, the PCI effect was already detectable after just 3 min of light exposure.

Although a tenfold higher concentration of immunotoxin was required in A549 cells compared to NCI-H1975, the difference in PCI efficacy was relatively modest. This suggests that, despite lower PD-L1 expression, prolonged incubation may facilitate non-specific, fluid phase endocytosis, contributing to the observed PCI effect.

### 3.6 Time-resolved flow cytometric analysis of anti-PD-L1 antibody uptake in NSLC cells

**Figure 6:**
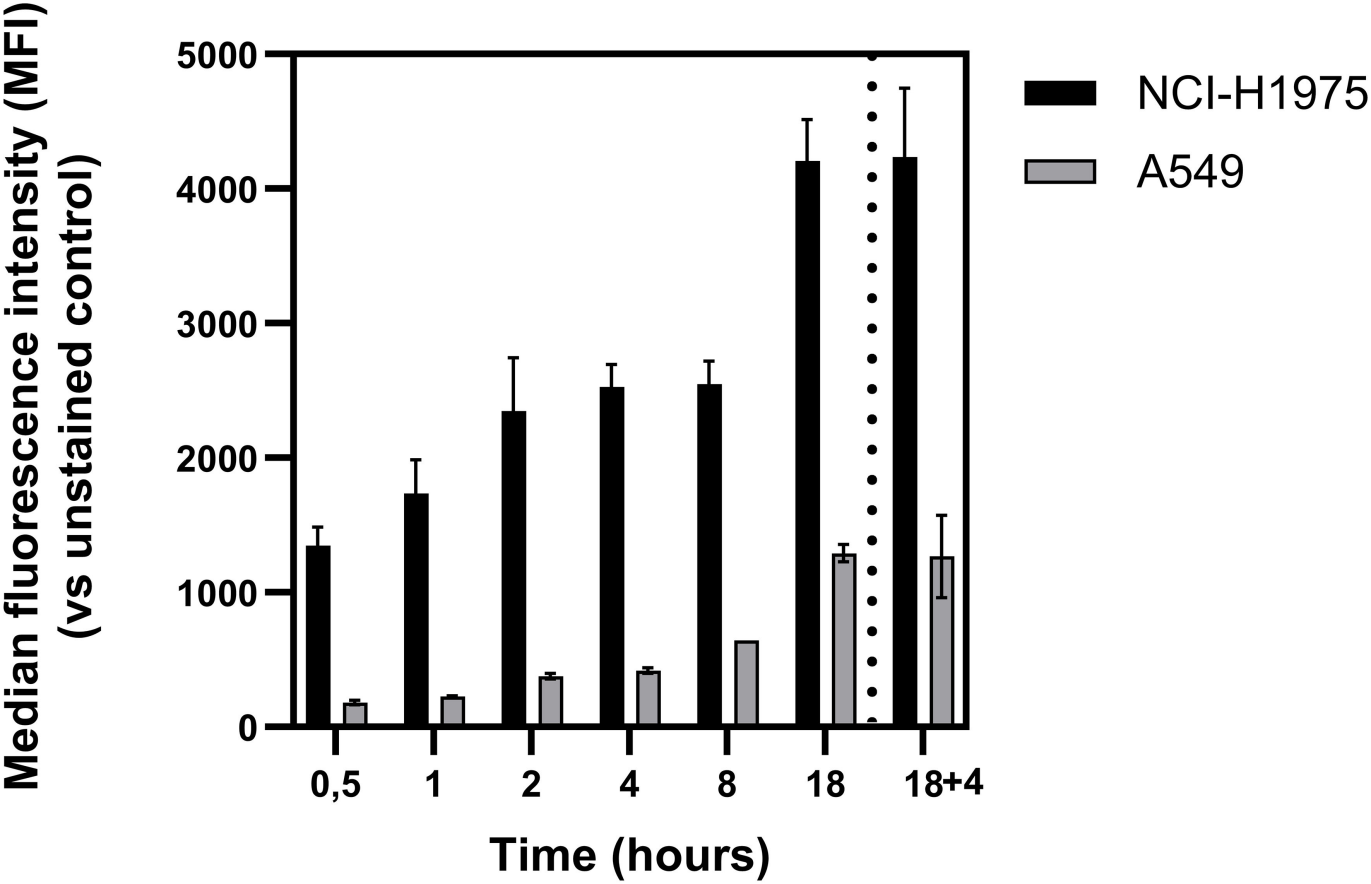
Flow cytometric quantification of PD-L1 uptake in NSCLC cell lines. PD-L1 expression levels were measured in NCI-H1975 (black bars) and A549 (grey bars) cells at the indicated time points using flow cytometry. Cells were stained with anti-PD-L1-Alexa647 antibody, and uptake was expressed as median fluorescence intensity (MFI) relative to unstained controls. The “18 + 4” condition refers to 18 hours of incubation followed by washout and an additional 4-hour chase in drug-free medium. Data represent mean ± SD from at least two independent experiments.

Flow cytometry was used to quantify the binding and uptake of the anti-PD-L1 antibody in NCI-H1975 and A549 NSCL cell lines at multiple time points, corresponding to those used in the confocal microscopy experiments. An additional condition, 18 h incubation followed by a 4 h chase, was included to mimic the Workflow 1 protocol and assess PD-L1 availability after washout. Median fluorescence intensity (MFI) revealed consistently higher levels of antibody-associated signal in NCI-H1975 cells compared to A549 cells across all time points, with differences spanning several orders of magnitude (**Figure 6**). These results confirm the characterization of NCI-H1975 as a PD-L1^high^ cell line and A549 as a PD-L1^low^ model. This binding profile aligns with the PCI efficacy data, where a 33-fold lower concentration of immunotoxin was sufficient to induce a potent PCI effect in NCI-H1975 cells after 18 h incubation compared to A549 cells. Notably, after just 4 h of incubation, NCI-H1975 cells already exhibited sufficient antibody uptake to support PCI activation, whereas A549 cells showed minimal fluorescence intensity, explaining the lack of a pronounced PCI effect during early illumination. Importantly, PD-L1-associated fluorescence remained stable following the 4 h chase period after 18 h incubation, with no significant reduction compared to continuous 18 h incubation. This suggests that the PD-L1-bound antibody remains bound to PD-L1 over time, supporting the feasibility of PCI protocols involving washout steps prior to light activation.

## 4 Discussion

In this study, we investigated the therapeutic potential of combining photochemical internalization (PCI) with the PD-L1-targeting immunotoxin anti-PD-L1-SAP in non-small cell lung cancer (NSCLC) models. To explore how PD-L1 expression levels influence treatment efficacy, we selected two NSCLC cell lines with distinct PD-L1 profiles: NCI-H1975 (PD-L1^high^) and A549 (PD-L1^low^), based on previously reported expression data (24–26). Our data clearly demonstrate that PCI-enhanced delivery of a PD-L1-targeting immunotoxin is a highly effective strategy for the cytotoxic targeting of PD-L1-positive NSCLC cells. In the PD-L1^high^ H1975 cell line, only picomolar concentrations of the immunotoxin were sufficient to induce a robust PCI-mediated cytotoxic response. This highlights the potency of the approach when target antigen expression is high. Interestingly, the PD-L1^low^ A549 cells also responded to PCI treatment, although a 10-fold higher concentration of the immunotoxin (within the nanomolar range) was required to achieve comparable effect. This suggests that even low levels of PD-L1 expression may be sufficient for PCI-mediated delivery, albeit with reduced efficiency, and underscores the importance of antigen density in determining therapeutic response. Reducing the immunotoxin incubation to 4 h in H1975 cells resulted in a marked decrease in potency, shifting the effective concentration to the nanomolar range. This suggests that sufficient cellular uptake and trafficking of the immunotoxin are essential for optimal PCI efficacy.

Confocal microscopy confirmed differential PD-L1 expression and trafficking of the PD-L1-targeting antibody. Alexa Fluor 647–labeled anti–PD-L1 antibody revealed markedly stronger fluorescence in NCI-H1975 cells compared to A549 cells, consistent with their higher PD-L1 expression. In PD-L1^high^ cells, the anti–PD-L1 antibody initially localized to the plasma membrane within 2 h, followed by gradual accumulation in lysosomal compartments, indicating receptor-mediated uptake and degradation of PD-L1. Flow cytometry confirmed significantly higher PD-L1 expression in NCI-H1975 cells compared to A549. Our observations are in line with Wong and Selbo (15), who reported that picomolar concentrations of the PD-L1-targeting immunotoxin were sufficient to induce strong cytotoxic responses in PD-L1^high^ breast cancer cells. In their study, a 4 h incubation with picomolar concentrations of the immunotoxin did not result in a PCI effect, which aligns with our observation that increasing the immunotoxin concentration to the nanomolar range was necessary to achieve effective PCI-mediated cytotoxicity under shorter incubation conditions. Notably, Alampi et al. similarly demonstrated differential intracellular routing of atezolizumab in NCI-H1975 and A549 cells, with more efficient lysosomal trafficking in A549 and evidence of recycling MVB (multivesicular bodies) involvement in H1975, supporting our conclusion that PD-L1 expression level and trafficking dynamics critically influence light-activated cytotoxic responses (14). Together, these results support the notion that while low concentrations are potent in PD-L1^high^ cells, successful PCI also depends on achieving a threshold concentration of internalized immunotoxin to trigger a robust cytotoxic response.

Taken together with previous studies, these results underscore the versatility and robustness of PCI as a platform for targeted cancer therapy. PCI-based strategies have now been successfully applied to a range of tumor-associated antigens, including EGFR (25, 26), CD133 (27), CD44 (28), and CSPG4 (29). The present study further expands this repertoire by demonstrating the efficacy of PD-L1-targeted PCI in NSCLC models. Collectively, this study and previous PCI investigations establish that PCI can reliably enhance intracellular delivery and cytotoxic potency across immunotoxins directed at multiple surface markers. These findings position PCI as a versatile, modular platform with strong potential for precision oncology applications.

Recent advances in PD-L1-targeted therapeutics further underscore the relevance of our findings. Yu et al. developed a dual-targeting nanobody-drug conjugate that combines PD-L1–specific nanobodies with a TLR7 agonist (SZU-101), enabling simultaneous PD-L1 binding and innate immune activation (30). Similarly, a novel PD-L1-targeting immunotoxin (D-CUS245C), in which the FDA-approved anti-PD-L1 antibody durvalumab was conjugated to cucurmosin, a type I RIP, was developed by Zhang et al. D-CUS245C exhibited strong cytotoxic activity at picomolar concentrations and delayed tumor growth in xenograft models. Confocal microscopy demonstrated that the durvalumab-FITC conjugate was specifically taken up by PD-L1-positive cells, with progressive cytoplasmic accumulation over 12 h, while PD-L1-negative cells showed no uptake (31). Additionally, conjugation of anti-PD-L1 nanoparticles with a truncated diphtheria toxin fragment significantly enhanced cytotoxicity in PD-L1-positive A431 cells compared to either components alone (32).

To our knowledge, only one study, Wong and Selbo (15) have demonstrated light-controlled elimination of PD-L1-positive cells using a photosensitizer-dependent endosomal escape mechanism (i.e., photochemical internalization). Taken together with our current findings, these studies reinforce the therapeutic potential of PD–L1 as a target for intracellular drug delivery. When viewed alongside PCI-based approaches described above, our study broadens and strengthens the concept of utilizing the PCI method for controlled targeting and elimination of PD-L1-positive cells in the TME. This growing body of evidence highlights PCI as a versatile and modular platform capable of enhancing the efficacy and specificity of targeted therapies across a range of tumor-associated antigens. We also show that PCI-based PD-L1-targeting is to some extent work in NSCLC cancer cells with low/ultralow expression of PD-L1.

To strengthen the translational relevance of our findings, future studies should include 3D tumor spheroid models to better mimic the TME and assess PCI efficacy under more physiologically relevant conditions. *In vivo* validation using xenograft or syngeneic mouse models is also essential to evaluate therapeutic efficacy, biodistribution, and safety. These models will help determine the clinical potential of PD-L1-targeted PCI and guide rational combination strategies with immunotherapies.

## 5 Conclusion

Our study demonstrates the *in vitro* efficacy and specificity of PCI-mediated delivery of the PD-L1 targeting immunotoxin anti-PD-L1-SAP in NSCLC cell lines with varying PD-L1 expression. Binding, uptake, and intracellular trafficking of the anti-PD-L1 mAb, revealed distinct dynamics between PD-L1^high^ NCI-H1975 and PD-L1^low^ A549 cells. PCI enabled cytotoxic effects correlating with PD-L1 expression and lysosomal accumulation of the PD-L1-targeting mAb. Co-treatment with atezolizumab reduced PCI efficacy in PD-L1^high^ cells highlighting the importance of receptor expression levels. The study also highlights that the intracellular trafficking behavior of the PD-L1-targeting mAb is a key determinant of PCI efficacy, underscoring the importance of antibody routing in addition to PD-L1 expression levels. These findings emphasize the role of target expression, intracellular trafficking, and treatment parameters in optimizing PCI. Overall, our work supports PD-L1 as a promising target for PCI-based precision drug delivery in NSCLC and warrants further preclinical evaluation.

## Data Availability

The original contributions presented in the study are included in the article/**Supplementary Material**. Further inquiries can be directed to the corresponding authors.
