# Supplementary material for: Photochemical enhancement of PD-L1-SAP immunotoxin efficacy in non-small cell lung cancer cell lines
Source: Front Immunol. 2026 Mar 13;17:1750003. doi: 10.3389/fimmu.2026.1750003 (PMC13021574; doi:10.3389/fimmu.2026.1750003)
Supplement: Supplementary file 1 [file Table1.docx]

**Supplementary Table 1**. P values and statistical tests. Analysis was performed using GraphPad Prism software.

| Figure | Test | N | Comparison | P value | Summary |
| --- | --- | --- | --- | --- | --- |
| 1E | Unpaired t test with Welch's correction | 3 | PDT 0.5 min vs. PCI of anti-PD-L1-SAP 1000 pM 0.5 min | 0.0001 | *** |
|  |  |  | PDT 1 min vs. PCI of anti-PD-L1-SAP 1000 pM 1 min | 0.0001 | *** |
|  |  |  | PDT 1.5 min vs. PCI of anti-PD-L1-SAP 1000 pM 1.5 min | 0.0004 | *** |
|  |  |  | PDT 2 min vs. PCI of anti-PD-L1-SAP 1000 pM 2 min | 0.0052 | ** |
|  |  |  | PDT 2.5 min vs. PCI of anti-PD-L1-SAP 1000 pM 2.5 min | 0.056 | ns |
|  |  |  | PDT 3 min vs. PCI of anti-PD-L1-SAP 1000 pM 3 min | 0.064 | ns |
| 1F | Unpaired t test with Welch's correction | 3 | PDT 0.5 min vs. PCI of anti-PD-L1-SAP 1000 pM 0.5 min | 0.0004 | *** |
|  |  |  | PDT 1 min vs. PCI of anti-PD-L1-SAP 1000 pM 1 min | 0.004 | ** |
|  |  |  | PDT 1.5 min vs. PCI of anti-PD-L1-SAP 1000 pM 1.5 min | <0.0001 | **** |
|  |  |  | PDT 2 min vs. PCI of anti-PD-L1-SAP 1000 pM 2 min | 0.0021 | ** |
|  |  |  | PDT 2.5 min vs. PCI of anti-PD-L1-SAP 1000 pM 2.5 min | 0.0056 | ** |
|  |  |  | PDT 3 min vs. PCI of anti-PD-L1-SAP 1000 pM 3 min | 0.0027 | ** |
| 1E | Unpaired t test with Welch's correction | 3 | PDT 0.5 min vs. PCI of anti-PD-L1-SAP 30 pM 0.5 min | 0.0272 | * |
|  |  |  | PDT 1 min vs. PCI of anti-PD-L1-SAP 30 pM 1 min | 0.0056 | ** |
|  |  |  | PDT 1.5 min vs. PCI of anti-PD-L1-SAP 30 pM 1.5 min | <0.0001 | **** |
|  |  |  | PDT 2 min vs. PCI of anti-PD-L1-SAP 30 pM 2 min | 0.0057 | ** |
|  |  |  | PDT 2.5 min vs. PCI of anti-PD-L1-SAP 30 pM 2.5 min | 0.0796 | ns |
|  |  |  | PDT 3 min vs. PCI of anti-PD-L1-SAP 30 pM 3 min | 0.0054 | ** |
| 1E | Unpaired t test with Welch's correction | 3 | PDT 0.5 min vs. PCI of anti-PD-L1-SAP 100 pM 0.5 min | 0.0076 | ** |
|  |  |  | PDT 1 min vs. PCI of anti-PD-L1-SAP 100 pM 1 min | 0.0133 | * |
|  |  |  | PDT 1.5 min vs. PCI of anti-PD-L1-SAP 100 pM 1.5 min | 0.0003 | *** |
|  |  |  | PDT 2 min vs. PCI of anti-PD-L1-SAP 100 pM 2 min | 0.0026 | ** |
|  |  |  | PDT 2.5 min vs. PCI of anti-PD-L1-SAP 100 pM 2.5 min | 0.0025 | ** |
|  |  |  | PDT 3 min vs. PCI of anti-PD-L1-SAP 100 pM 3 min | 0.0236 | ** |
| 1G | Unpaired t test with Welch's correction | 3 | PDT 0.5 min vs. 4h PCI of anti-PD-L1-SAP 1000 pM 0.5 min | 0.0003 | *** |
|  |  |  | PDT 1 min vs. 4h PCI of anti-PD-L1-SAP 1000 pM 1 min | 0.0107 | * |
|  |  |  | PDT 1.5 min vs. 4h PCI of anti-PD-L1-SAP 1000 pM 1.5 min | <0.0001 | **** |
|  |  |  | PDT 2 min vs. 4h PCI of anti-PD-L1-SAP 1000 pM 2 min | 0.0003 | *** |
|  |  |  | PDT 2.5 min vs. 4h PCI of anti-PD-L1-SAP 1000 pM 2.5 min | 0.0024 | ** |
|  |  |  | PDT 3 min vs. 4h PCI of anti-PD-L1-SAP 1000 pM 3 min | 0.0108 | * |
| 1G | Unpaired t test with Welch's correction | 3 | PDT 0.5 min vs. 4h PCI of anti-PD-L1-SAP 1000 pM + Atez. 0.5 min | 0.0108 | * |
|  |  |  | PDT 1 min vs. 4h PCI of anti-PD-L1-SAP 1000 pM + Atez. 1 min | 0.3457 | ns |
|  |  |  | PDT 1.5 min vs. 4h PCI of anti-PD-L1-SAP 1000 pM + Atez. 1.5 min | 0.0322 | * |
|  |  |  | PDT 2 min vs. 4h PCI of anti-PD-L1-SAP 1000 pM + Atez. 2 min | 0.011 | * |
|  |  |  | PDT 2.5 min vs. 4h PCI of anti-PD-L1-SAP 1000 pM + Atez. 2.5 min | 0.0123 | * |
|  |  |  | PDT 3 min vs. 4h PCI of anti-PD-L1-SAP 1000 pM + Atez. 3 min | 0.037 | * |
| 1H | Unpaired t test with Welch's correction | 3 | PDT 0.5 min vs. 4h PCI of anti-PD-L1-SAP 1000 pM 0.5 min | 0.3119 | ns |
|  |  |  | PDT 1 min vs. 4h PCI of anti-PD-L1-SAP 1000 pM 1 min | 0.0566 | ns |
|  |  |  | PDT 1.5 min vs. 4h PCI of anti-PD-L1-SAP 1000 pM 1.5 min | 0.005 | ** |
|  |  |  | PDT 2 min vs. 4h PCI of anti-PD-L1-SAP 1000 pM 2 min | 0.0009 | *** |
|  |  |  | PDT 2.5 min vs. 4h PCI of anti-PD-L1-SAP 1000 pM 2.5 min | 0.0008 | *** |
|  |  |  | PDT 3 min vs. 4h PCI of anti-PD-L1-SAP 1000 pM 3 min | 0.0064 | ** |
| 1H | Unpaired t test with Welch's correction | 3 | PDT 0.5 min vs. 4h PCI of anti-PD-L1-SAP 1000 pM + Atez. 0.5 min | 0.2359 | ns |
|  |  |  | PDT 1 min vs. 4h PCI of anti-PD-L1-SAP 1000 pM + Atez. 1 min | 0.2279 | ns |
|  |  |  | PDT 1.5 min vs. 4h PCI of anti-PD-L1-SAP 1000 pM + Atez. 1.5 min | 0.0993 | ns |
|  |  |  | PDT 2 min vs. 4h PCI of anti-PD-L1-SAP 1000 pM + Atez. 2 min | 0.0612 | ns |
|  |  |  | PDT 2.5 min vs. 4h PCI of anti-PD-L1-SAP 1000 pM + Atez. 2.5 min | 0.0529 | ns |
|  |  |  | PDT 3 min vs. 4h PCI of anti-PD-L1-SAP 1000 pM + Atez. 3 min | 0.0489 | * |
