# Supplementary material for: Photochemical enhancement of PD-L1-SAP immunotoxin efficacy in non-small cell lung cancer cell lines
Source: Front Immunol. 2026 Mar 13;17:1750003. doi: 10.3389/fimmu.2026.1750003 (PMC13021574; doi:10.3389/fimmu.2026.1750003)
Supplement: Supplementary file 3 [file DataSheet2.pdf]

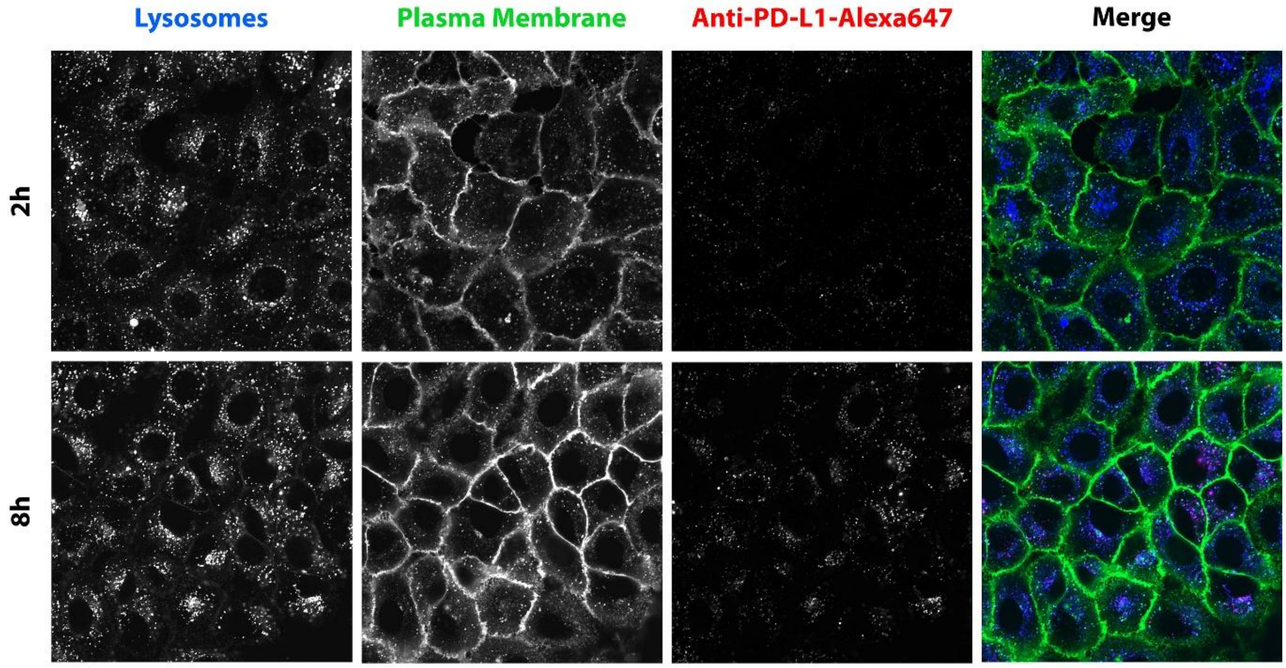

Supplementary Figure 2. Intracellular localization and trafficking of anti-PD-L1-AlexaFluor647 in A549 cells. Representative photomicrographs of 2 h and 8 h are shown. Anti-PD-L1-biotinylated conjugated with Alexa 647-Streptavidin (red) were used at dilution 1:400 and 1:265. LysoTracker Blue DND-22 (0.4 µM. blue) and CellMask Green Plasma Membrane Stain (0.1×, green) were added and incubated with cells for 20 min at 37 °C. Photomicrographs were taken using laser scanning confocal microscope. Scale bar: 20µm.
